# Short term effects of a commercial post workout product, Adenoflex^®^, on hematocrit levels and muscular endurance

**DOI:** 10.1186/1550-2783-10-S1-P10

**Published:** 2013-12-06

**Authors:** Patrick L Jacobs

**Affiliations:** 1 Superior Performance Research, Miami FL 33186, USA

## Background

Few post-workout products have been properly investigated in finished commercial form. This study was carried out in order to determine the short term (14 days) effects of Adenoflex^®^ (World Health Products, LLC; Stamford, CT) on hematocrit levels and measures of muscular endurance.

## Methods

Twelve recreationally active men, 28.5 ± 5 years of age and 197.1 ± 32.4 pounds body weight, took part in this double-blind, placebo-controlled trial on a volitional basis. Study participants were randomly assigned to receive either Adenoflex (AD) or Placebo (PL) for a 14 day period and were directed to take two servings per day for the first 8 days (immediately after training and five hours following) and one serving daily for the final 6 days (immediately after training). All participants completed a testing series prior to and following the supplementation period including measurement of hematocrit levels and upper extremity muscular endurance. Hematocrit was determined using standard procedures by centrifuging blood in a microhematocrit tube in a microhematocrit centrifuge. Muscular endurance was determined by performance of three sets of bench press and three sets of barbell curls with bodyweight and 1/3 bodyweight, respectively, with one minute recovery periods between sets. Work volume was calculated as repetitions completed times resistance utilized with work volume and reps completed examined per each set completed and as total values for bench press and barbell curls. Pre- and post-supplementation values of all variables were standardized into change scores relative to baseline values. Statistical analyses were conducted using one-way ANOVAs with the accepted level of significance set at p < 0.05.

## Results

Results indicated that while AD produced a mean increase of hematocrit from 43.67% to 45.83% and PL did not change (pre and post = 43.83%) these differences within and between groups were not statistically significant. Bench press repetitions change scores for the three sets were (+3.0, +1.3, +1.0) for AD and (+1.7, -0.7, -0.2) with PL. No significant differences were detected between conditions for reps completed or total work volume per set. However, the increase in total bench reps completed with AD (+5.3) was statistically greater than with PL (+0.8) (p=0.05) and the total bench press work volume change scores were also statistically different between conditions (AD=+883.3; PL=+212.5 rep lbs). Mean change scores for the three sets of barbell curls were (+2.8, +4.2, +4.0) with AD were not significantly different from PL (+0.8, +2.6, +0.7) with no significant differences detected between conditions in work volume per set (p’s>0.05). While not statistically significant, AD produced a mean increase of 11.0 total BC reps compared with 4.0 reps increase with PL.

## Conclusion

These findings indicate that upper extremity muscular endurance is significantly enhanced with Adenoflex^®^. This may indicate an improved training stimulus for muscular endurance and/or for muscular hypertrophy.

## Funding

This study was supported by funding from World Health Products, LLC; Stamford, CT, USA.

